# Alterations of long non-coding RNA and mRNA profiles associated with extracellular matrix homeostasis and vascular aging in rats

**DOI:** 10.1080/21655979.2021.1889129

**Published:** 2021-02-28

**Authors:** Qianqin Li, Zezhou Xiao, Yongsheng Wang, Ximao Liu, Hao Liu, Zhiwen Luo, Shaoyi Zheng

**Affiliations:** Department of Cardiovascular Surgery, Nanfang Hospital, Southern Medical University, Guangzhou Guangdong, China

**Keywords:** Long non-coding RNA, mRNA, rna sequencing, vascular aging

## Abstract

Vascular aging has been closely associated with various cardiovascular disorders; however, its molecular mechanism remains poorly understood. In our study, RNA sequencing was utilized to explore the expression profiles of long non-coding RNAs (lncRNAs) and mRNAs in the thoracic aortas of young (3 weeks) and old (16 weeks) rats. Functional categorization of differentially expressed mRNAs was evaluated using the Gene Ontology and Kyoto Encyclopedia of Genes and Genomes databases, and lncRNA–microRNA–mRNA networks was constructed using Cytoscape software. In addition, three upregulated and three downregulated lncRNAs were further confirmed by quantitative reverse transcriptase-polymerase chain reaction. A total of 36 lncRNAs and 922 mRNAs were differential expression in the thoracic aortas of young and older rats. In addition, we found differentially expressed mRNAs that were enriched in multiple biological processes and signaling pathways associated with angiogenesis, such as extracellular matrix–receptor interaction and adenosine 3ʹ,5ʹ-monophosphate-activated protein kinase (AMPK) signaling. Moreover, AABR07013558.1, AABR07014823.1, and AABR07031489.1 were upregulated and ABR07053849.3, AABR07067310.2, and AC111292.1 were downregulated in the thoracic aortas of older rats compared with the young ones. Therefore, our findings provide several potential lncRNAs and mRNAs and signaling pathways related to vascular aging, which provide new clue for underlying the improvement of vascular aging.

## Graphical Abstract

**Figure:**
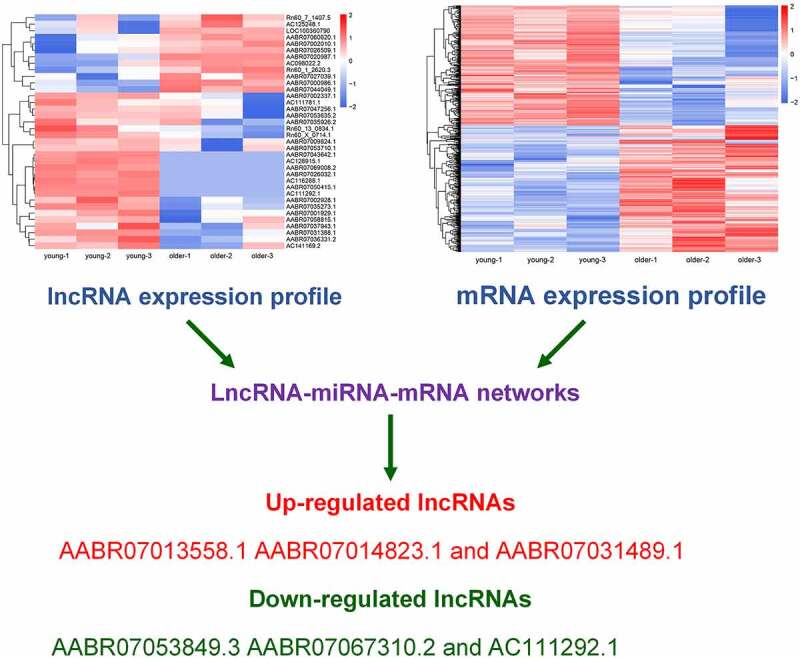

## Introduction

Vascular aging refers to the dysfunctional alterations of blood vessels that occur during the aging process. Its common features are structural and functional changes, such as dysregulation of vascular homeostasis and vascular dyshomeostasis, enhanced vascular cell senescence, and vascular remodeling [1]. In contrast to young vessels, aging blood vessels usually exhibit diffused stiffness, thickened arterial walls, luminal dilation, endothelial cell injury, fibrosis, and calcification [1,2]. During the past decades, it has been well-established that vascular aging is critically involved in the pathogenesis of various human diseases, such as cardiovascular disorders (CVD), hypertension, atherosclerosis, stroke, coronary heart disease, and other aging-related disorders [2–4]. For instance, arterial stiffness substantially contributes to the high mortality and CVD events in patients with chronic kidney disease as well as to the exercise intolerance of patients with heart failure [5]. The delay of vascular aging through physical training and food additives, including vitamins and resveratrol, has been trialed as a promising strategy for preventing aging-related CVD and cerebrovascular diseases [6]. The prevalent pathogenic implications of vascular aging suggest that a complete understanding of vascular aging mechanisms would greatly facilitate the prevention and treatment of aging-related diseases.

Previous reports showed that the progression of aging in blood vessels is mediated by complex networks of cellular and molecular events [7,8]. For instance, the elevated reactive oxygen species produced by the mitochondria during aging exerted critical roles in the stiffening of the large elastic aorta and vascular endothelial dysfunction [7,9]. Moreover, the age-related activation of inflammatory factors, including chemokines and cytokines, was shown to be closely associated with endothelial senescence and vascular aging [7,10]. In addition, vascular aging is also mediated by the reduced ability of responsiveness to molecular stresses, impaired maintenance of proteostasis, DNA damage and genomic instability, vascular endothelial and smooth muscle cell senescence, and dysregulations of autophagy, nutrient-sensing pathways, and extracellular matrix (ECM)-remodeling [7,11]. Of note, recent investigations demonstrated that epigenetic regulation also plays essential roles in the aging of blood vessels, mediated by alterations of DNA methylation pattern, histone post-translational modifications, non-coding RNAs, and chromatin remodeling [1,2,7]. As shown by a recent report, the trimethylation of histone H3 at K79 in the promoter region of the Sirtuin-3 gene induced by the activation of adenosine 3ʹ,5ʹ-monophosphate-activated protein kinase (AMPK) could prevent age-associated vascular dysfunction through the regulation of mitochondrial biogenesis and senescence [12]. However, the epigenetic landscape underlying vascular aging progression still remains poorly elucidated.

Long non-coding RNAs (lncRNAs) are a recently characterized group of non-coding RNAs, usually comprising >200 nucleotides, and are recognized as essential components of epigenetic regulation system [13,14]. Extensive research has shown that lncRNAs perform vital regulatory roles in various biological and pathogenic processes, mainly due to their potent ability of modulating gene expression at both transcriptional and post-transcriptional levels, such as sponging microRNA, regulating RNA splicing and mRNA processing, and acting as cis-acting components [15]. Moreover, lncRNAs have been characterized as key regulators of endothelial and vascular smooth cell function and homeostasis and respond to blood vessel wall injuries, thus being essentially involved in the pathophysiology of various vascular disorders [16]. For instance, the antisense non-coding RNA in the INK4 locus (lncRNA ANRIL) could promote atherosclerosis pathogenesis, possibly mediated by the regulation of the inflammatory pathways, and is considered a risk factor for atherosclerosis-related vascular diseases [17]. More importantly, lncRNAs have been implicated in cellular processes closely associated with vascular aging [18,19]. The senescence of vascular smooth muscle cells was recently reported to be regulated by ANRIL and lncRNA-ES3 [18,19], and vascular endothelial cell aging and senescence could be modulated by lncRNAs H19 and ASncmtRNA-2 [20,21]. These recent investigations indicated the essential roles of lncRNA in vascular aging and related pathogenesis, which are worthy of further investigations. In addition, studies confirmed that lncRNA can affect the growth and metabolism of organisms by regulating changes in the transcriptome. Therefore, further investigation of the roles of lncRNAs in the whole transcriptome and their relationship with mRNAs can provide clues for the prevention, delay, and treatment of vascular aging.

In this study, we explored characterization of the differentially expressed lncRNAs and mRNAs for the first time in the thoracic aortic tissues of young and older rats by high-throughput RNA deep sequencing. The results may provide new insights into molecular mechanism driving vascular aging, which might be further explored as a basis to develop new treatments for aging-related vascular disorders.

## Material and methods

### Animals and grouping

Healthy male Sprague Dawley (SD) rats were purchased from Chengdu Dashuo Biotechnology Co., Ltd. (Chengdu, China) and maintained in a pathogen-free barrier facility at 27°C at the Laboratory Animal Center of the Nanfang Hospital of Southern Medical University. All experimental procedures on SD rats in this study was approved in advance by the Experimental Animal Care and Use Committee of the Nanfang Hospital, Southern Medical University and performed following the Guide for the Care and Use of Laboratory Animals (National Institutes of Health, 1996). To explore the lncRNA and mRNA expression profiling related to vascular aging, three healthy male rats aged 3 weeks with an average body weight of 52 g were used as the young group and another three healthy male rats aged 16 weeks with a body weight of approximately 480 g were selected as the older group. When SD rats reached the humane end point of euthanasia, they were euthanized in the euthanasia chamber with 20% chamber volume/min of CO_2_ [22]. Death was then confirmed after cervical dislocation.

### Vascular tissue collection and RNA extraction

SD rats were sacrificed at designated ages through anesthesia with chloral hydrate, and the rat thoracic aortic tissues were surgically collected and immediately stored in liquid nitrogen. The extraction of total RNA from rat aortic tissues for RNA sequencing was performed using Invitrogen™ TRIzol™ Reagent (#15,596,026; Thermo Fishier Scientific) following the manufacturer’s instructions. In brief, approximately 0.1 g rat aortic tissues were homogenized in 1 mL TRIzol™ Reagent with a homogenizer, which was then incubated with 0.5 mL isopropanol for 10 min at 4°C, followed by centrifugation for 10 min at 12,000 × *g* at 4°C. The resulting pellet was washed with 1 mL 75% ethanol and resuspended in 50 µL RNase-free water. The RNA samples were analyzed by NanoDrop™ 1000 spectrophotometer (Thermo Fishier Scientific), and samples with a A260/A280 ratio of ~2 were stored at −80°C for subsequent assays.

### Deep sequencing and quantitation

The quantitation of lncRNA and mRNA expression profiles in rat aortic tissues were performed by RNA deep sequencing as previously described with minor modifications [20]. In brief, the total RNA samples, prepared as mentioned above, were first subjected to ribosomal RNA (rRNA) component removal using Ribo-zero rRNA Removal Kit (Human/Mouse/Rat) (Epicentre, USA) as instructed by the manufacturer. Subsequently, RNA-seq libraries were established using approximately 2.5 µg RNA samples of each group using NEBNext Ultra II Directional RNA Library Prep Kit for Illumina (#E7420S; NEB, USA) according to the manufacturer’s instructions. The established libraries were then subjected to deep sequencing using Illumina Hiseq 4000 sequencing platform (Illumina, CA, USA). For selection of clean reads, raw reads from sequencing were then submitted to the in-house Perl scripts to delete low-quality reads and those containing poly-N and adapters. All clean reads of lncRNA and mRNA molecules in each sample were then used for the calculation of values of expected number of fragments per kilobase of transcript sequence per million base pairs sequenced (FPKM) through the statistical analysis provided by Cuffdiff software. The significant differentially expressed lncRNA and mRNA of the young and older rat groups were defined by the combination of fold change (≥2) and P-value (<0.05).

### Bioinformatic analysis

The numbers of lncRNAs within different size ranges and encoded by DNA sequences on each rat chromosome were counted separately. Hierarchical clustering of lncRNA or mRNA differential expression was presented using heatmaps created in R language software. Moreover, the differential expression of mRNA of the young and older rat groups was also evaluated using a scatter plot constructed using the transcripts per million (TPM) values and a false discovery rate of <0.01. Functional categorization of differentially expressed mRNA of the young and older rat groups was performed using the Database for Annotation, Visualization and Integrated Discovery system based on the following Gene Ontology (GO) terms: molecular functions, biological processes, and cellular components. Moreover, the signaling pathway enrichments of these differentially expressed mRNAs were assessed using the Kyoto Encyclopedia of Genes and Genomes (KEGG) database (http://www.genome.jp/kegg/). Finally, the interaction networks between lncRNA, microRNAs, and mRNAs were constructed using Cytoscape software.

### LncRNA expression validation

The differential expression of lncRNAs determined using deep sequencing was further confirmed by quantitative reverse transcriptase-polymerase chain reaction (qRT-PCR) method. In brief, total RNA samples were extracted using Invitrogen™ TRIzol™ Reagent (#15,596,026; Thermo Fishier Scientific) as described above. The cDNA library was then established using TransScript® Reverse Transcriptase kit (#AT101-02; TransGene Biotech, Beijing, China) as instructed by the manufacturer. Subsequently, the qRT-PCR assay was then performed to measure the expression levels of lncRNA between groups using the TransStart® Green qPCR SuperMix (#AQ101-01; TransGene Biotech, Beijing, China) following the manufacturer’s instructions. The relative expression levels of lncRNAs were finally calculated via the 2^−ΔΔCt^ method combined with normalization to the expression levels of glyceraldehyde-3-phosphate dehydrogenase). Primers used for the qRT-PCR method are provided in (Table 1).

**Table 1.** Primers in this study

Primer Name	Sequence (5’ to 3’)
AABR07013558.1-F	TCGTGACCCTCCTACCTCAC
AABR07013558.1-R	CCTGGTTCACAACCTCACCT
AABR07014823.1-F	GCTTGGCAGATGGGTTAAGA
AABR07014823.1-R	GTCTGGTTTGGTCCCACTGT
AABR07031489.1-F	TTACGAGCATGTTGGCTCAG
AABR07031489.1-R	TTTGGTGCCACCTAGGAAAG
AABR07053849.3-F	GATGGATTGTTGTGCTGTCAA
AABR07053849.3-R	CCCAAACACTTTGGCAAATA
AABR07067310.2-F	GAGCACTCTCTGGGGTTCAG
AABR07067310.2-R	TGACGTCACATCCTGCTCTC
AC111292.1-F	TAGTAGGCCCGAAAGAAGCA
AC111292.1-R	ATGTCCCCTCAAGCATTCAC
GAPDH-F	GAGTCAACGGATTTGGTCG
GAPDH-R	GAGTCAACGGATTTGGTCGT

### Statistical analysis

All experiments were performed with at least three replicates. Quantitative data were expressed as means ± standard deviation and analyzed using SPSS 18.0 software. Differences between the two groups were evaluated using the Student’s t-test, and statistically significant differences were defined by a P value of <0.05.

## Results

LncRNAs perform vital regulatory roles in various biological and pathogenic processes due to their potent ability of modulating gene expression at both transcriptional and post-transcriptional levels, such as sponging microRNA, regulating RNA splicing, mRNA processing, and acting as cis-acting components. In this study, we explored characterization of the differentially expressed lncRNAs and mRNAs in the thoracic aortic tissues of young and old rats for the first time. Then, GO and KEGG analyses were performed for the differentially expressed mRNAs, and lncRNA–microRNA–mRNA interaction networks were established based on the differentially expressed lncRNAs and mRNAs. Finally, six lncRNAs in the networks were verified to test the reliability of RNA sequencing.

### Significant differential lncRNA profiles in thoracic aortas of young and older rats

**Figure 1.**
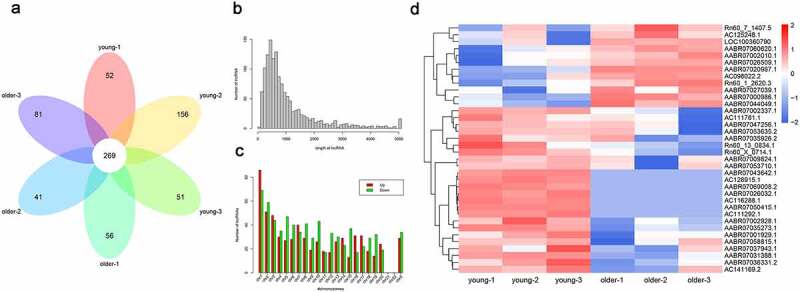
**Differential lncRNAs profiles in thoracic aortas of young and older rats**. (a) A Venn diagram showing the numbers of lncRNAs characterized in the young and older rat’ thoracic aortas. Using RNA deep sequencing, a total of 269 common lncRNAs were identified in each group. (b) The size distribution of lncRNAs identified in the rat’ thoracic aortas by RNA sequencing. (c) The genomic location of lncRNAs significantly differentially expressed in the thoracic aortas of young and older rats. Upregulated and downregulated lncRNAs in older rats compared to those of young were shown in red and green colors, respectively. (d) Hierarchical clustering of differentially expressed lncRNAs between the young and older rat’ thoracic aortas. The alterations in the expression of lncRNAs are presented as heatmaps, in which high and low expressions of lncRNAs were shown in red and blue colors, respectively. lncRNAs: long non-coding RNAs; chr: chromosome

To explore the potential involvements of lncRNAs in vascular aging, we performed a general characterization of lncRNAs that were differentially expressed in the thoracic aortas of the young (3-week-old) and older (16-week-old) rats by deep sequencing, with three biological replicates in each group (Figure 1a). Our transcriptional analysis showed that 269 lncRNAs in total were characterized in each replicate for both young and older rats (Figure 1a). The sizes of these identified lncRNAs ranged between 100 and nearly 5,000 bp, and the majority of these lncRNAs were of sizes between 200 and 1,000 bp (Figure 1b). In addition, we showed that these lncRNAs were encoded by genomic sequences located on most rat chromosomes, except for chromosome #21 and chromosome #22 (Figure 1c). The rat chromosome #1 was shown to encode the largest number of differentially expressed lncRNAs in the young and older rats’ thoracic aortas (Figure 1c). Moreover, our hierarchical clustering analysis of these significantly expressed lncRNAs revealed significant differences in lncRNA expression profiles of the young and older rat’ thoracic aortas (Figure 1d). In particular, 36 total lncRNAs were significantly differentially expressed, including 24 upregulated and 12 downregulated lncRNAs in the aortas of young rats compared with those of older ones (Figure 1d). The large number of differentially expressed lncRNAs of the young and older rat’ thoracic aortas suggested the potential functions of lncRNAs during the vascular aging processes.

### Differential gene expression profiles in thoracic aortas of the young and older rats

**Figure 2.**
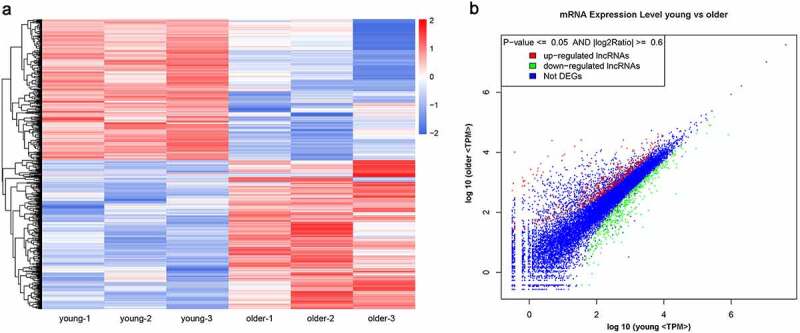
**Significantly altered mRNA profiles in thoracic aortas of young and older rats**. (a) Hierarchical clustering of differentially expressed mRNAs in the thoracic aortas of the young and older rats. mRNAs with high and low expression are shown in red and blue colors, respectively. (b) A scatter plot presenting the expression changes in mRNAs of the young and older rats’ thoracic aortas. mRNAs with upregulated, downregulated, and similar expressions in the young rats compared with older ones are shown in red, green, and blue colors, respectively. DEG: differentially expressed genes; TPM: transcripts per million

For insights into the molecular mechanisms underlying vascular aging progression, we also characterized the significantly differentially expressed mRNAs in the thoracic aortas of young and older rats during RNA deep sequencing. The expression levels of 922 mRNAs were significantly altered between the young and older rat’ thoracic aortas, including 474 upregulated and 448 downregulated mRNAs in the thoracic aortas of older rats compared with those of young rats (Figure 2a; Supplemental Table 1). Through hierarchical clustering based on these differentially expressed mRNAs, we revealed significantly distinct gene expression profiles in the thoracic aortas of young and older rats (Figure 2a). In addition, the scatter plot established on all identified genes showed that a large number of genes showed remarkably altered expression levels between the young and older rat’ thoracic aortic tissues (Figure 2b). More detailed information of these differentially expressed genes is listed in Supplemental Table 1. The marked alterations of gene expression profiles between these young and older rats’ thoracic aortas indicated that complex molecular mechanisms and signaling pathways are associated with the vascular aging process.

### Functional categorization of differentially expressed genes of young and older rats’ thoracic aortas

**Figure 3.**
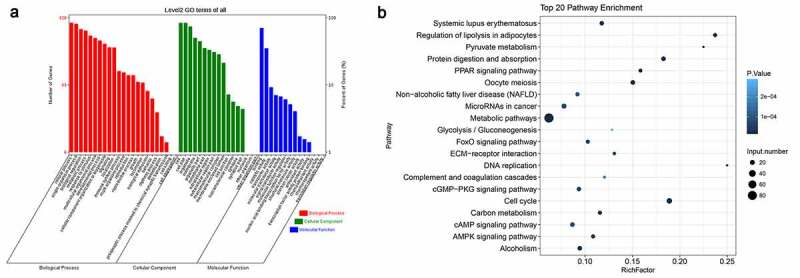
**Functional categorization of significantly altered mRNAs between young and older rats’ thoracic aortas**. (a) Categorization of differentially expressed mRNAs between young and older rat’ thoracic aortas using GO terms. (b) The KEGG enrichment signaling pathways of differentially expressed genes between young and older rat’ thoracic aortas. GO: gene ontology; KEGG: Kyoto Encyclopedia of Genes and Genomes

To obtain a comprehensive view of biological processes possibly linked with vascular aging, we functionally categorized the differentially expressed mRNAs in the thoracic aortic tissues of young and older rats. Based on GO analysis of biological processes, we found that these differentially expressed genes were significantly enriched in single-organism process, biological regulation, metabolism, responses to stimulus, development, cellular component organization, signaling, localization, the immune system, reproduction, growth, locomotion, cell killing, biological adhesion, and others (Figure 3a). In addition, proteins encoded by these differential genes were predicted to be distributed in many subcellular locations, such as the membrane, the extracellular region, macromolecular complex, the membrane-enclosed lumen, cell junction, synapse, and the nucleoid (Figure 3a). Moreover, differentially expressed genes have variable molecular functions, including binding, catalysis, transport, molecular transduction, signal transduction, transcription factor, structure molecules, electron carrier, antioxidant, chemoattractant, and translation regulator, suggesting great cellular processes alterations during vascular aging (Figure 3a).

In addition, we further analyzed the enrichment of these differentially expressed mRNAs in KEGG signaling pathways (Figure 3b). Using KEGG database, we found that differentially expressed genes were remarkably enriched in cell cycle processes, ECM–receptor interaction, cyclic adenosine 3ʹ,5ʹ-monophosphate (cAMP) signaling, cyclic guanosine monophosphate and the dependent protein kinase (cGMP-PKG) signaling, AMPK signaling, peroxisome proliferator-activated receptor (PPAR) signaling and other signaling pathways, which are associated with angiogenesis and vascular aging. The significant enrichments of these differentially expressed genes in various signaling pathways further suggest that alterations in cellular signaling events underlie the vascular aging processes.

### Gene expression networks

**Figure 4.**
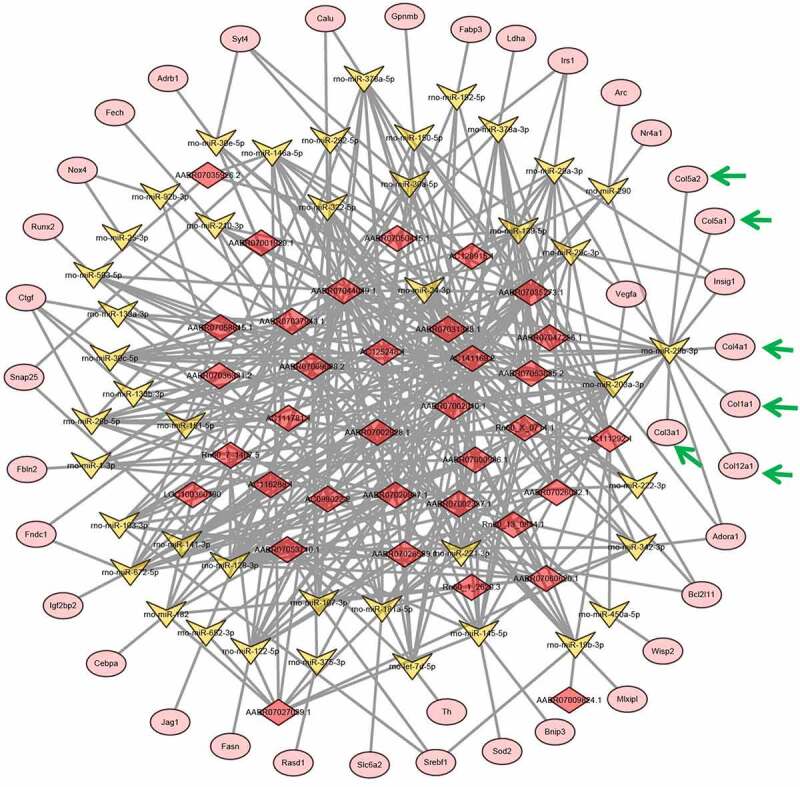
**lncRNA–microRNA–mRNA networks**. lncRNA–microRNA–mRNA networks were constructed using Cytoscape software base on the differentially expressed lncRNAs and mRNAs identified in the young and older rat’ thoracic aortas by RNA deep sequencing. LncRNAs, microRNAs, and mRNAs are indicated by red diamonds, yellow irregular quadrilaterals, and pink ovals, respectively. Predicted interactions between them are shown by gray lines. The mRNAs involved in collagen synthesis are highlighted with green arrow. Detailed information is provided in Supplemental Table 2

Previous reports showed that lncRNAs could induce gene expression alterations by sponging microRNAs [23]. For a deeper understanding of the vascular aging mechanism, we established lncRNA–microRNA–mRNA interaction networks using bioinformatic prediction based on abovementioned lncRNAs and mRNAs that were differentially expressed in young and older rat’ thoracic aortas. Using this bioinformatic analysis, we showed that a large set of microRNAs were predicted to interact with both the differentially expressed lncRNA and mRNAs between the young and older rat’ thoracic aortas, as discovered by deep sequencing (Figure 1d and Figure 4; Supplemental Table 1 and Table 2). Importantly, we showed that many collagen-encoding genes were predicted to be targeted by several microRNAs, which were also predicted to be sponged by several lncRNAs that were differentially expressed between the young and older rat’ thoracic aortas (Figure 4). For instance, lncRNA AC111292-1 was predicted to sponge mo-miR-29b-3p, mo-miR-222-3p, mo-miR-29 c-3p, mo-miR-29a-3p, and other microRNAs (Figure 4). Among the microRNAs sponged by AC111292-1, the mo-miR-29b-3p was shown to target multiple collagen genes including collagen 5A2 (Col5a2), Col5a1, Col4a1, Col1a1, Col3a1, and Col12a1 (Figure 4). These results suggest that the regulation of collagen synthesis by lncRNAs through sponging microRNAs may serve as an important regulator of vascular aging.

### Validation of differential expression lncRNA in young and older rat’ thoracic aortas

**Figure 5.**
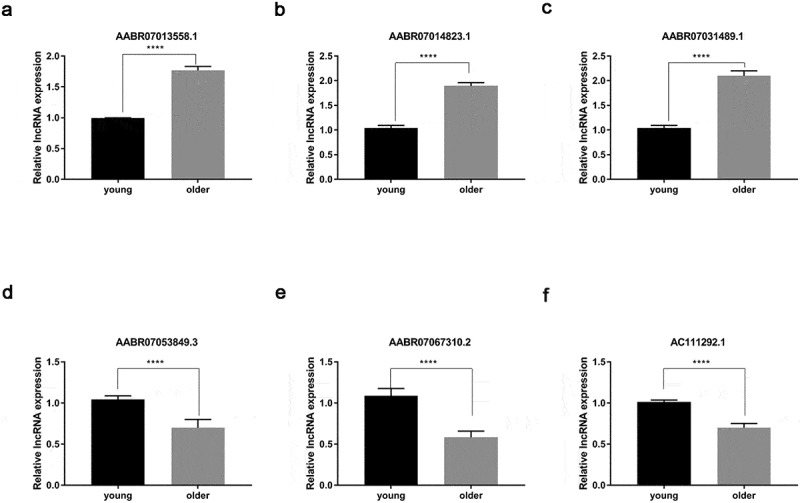
**Verification of differential expression of representative lncRNAs between young and older rat’ thoracic aortas**. The upregulated expression levels of AABR07013558.1 (a), AABR07014823.1 (b), and AABR07031489.1 (c) as well as the downregulated expression of AABR07053849.3 (d), AABR07067310.2 (e), and AC111292.1 (f) in the thoracic aortas of older rats compared to young ones were assessed using the qRT-PCR method. At least three biological replicates were performed, GAPDH as the internal standard. lncRNAs: long non-coding RNAs; ****P < 0.0001

To test the reliability of our RNA sequencing results, we analyzed the expression of six lncRNAs by qRT-PCR method, which were shown to be differentially expressed in the thoracic aortas of young and older rats (Figure 5a, Figure 5b, Figure 5c, Figure 5d, Figure 5e, and Figure 5f). Among them, we found that the expression levels of AABR07013558.1, AABR07014823.1, and AABR07031489.1 in the thoracic aortas of older rats were significantly elevated compared with the levels in the thoracic aortas of young rats (Figure 5a, Figure 5b, Figure 5c). Meanwhile, the expression of three other lncRNAs, AABR07053849.3, AABR07067310.2, and AC111292.1 were remarkably decreased in the thoracic aortas of older rats compared with that in the thoracic aortas of young rats (Figure 5d, Figure 5e, and Figure 5f). The alterations in the expression of these six lncRNAs by the qRT-PCR method were all consistent with the RNA sequencing results (Figure 1d). These results showed the reliability of the differential expression of lncRNAs and mRNAs as characterized by RNA deep sequencing in this study, which could serve as a solid basis for further functional investigations of lncRNAs and mRNAs in vascular aging processes.

## Discussion

Molecular mechanisms underlying vascular aging have been a major research interest during the past decades because of the high incidences and mortalities caused by CVD, atherosclerosis, and aging-related pathogenic conditions [1,3,4,24]. Despite the well-recognized strong association between aging and vascular dysfunctions, molecular mechanisms driving the blood vessel deregulation during aging still remain poorly understood. In the present study, we performed a global identification of lncRNAs and mRNAs with significantly different expression in the thoracic aortas of 3-week-old young rats and 16-week-old older rats using the next-generation RNA sequencing method. We demonstrated that a group of 36 lncRNAs that were significantly differentially expressed in older rats’ thoracic aortic tissues compared with young rats, indicating the potential mediating roles of lncRNAs-mediated gene expression alterations in the aging of vascular systems. Moreover, we identified a large number of mRNAs showing remarkable expression changes in older rats’ aortas, which were greatly enriched in various biological processes and signaling pathways closely associated with angiogenesis. In addition, we showed by bioinformatic prediction that these differentially expressed lncRNAs may cause gene expression alterations during aging progression by sponging many microRNAs, which could specifically target the expression of several collagen genes. These transcriptome sequencing data combined with bioinformatic tools disclosed a new paradigm of blood vessel aging and dysfunction that are mediated by epigenetic regulations. Therefore, these data may provide new insights into the molecular mechanism underlying vascular aging, which could be explored for preventing and treating vascular disorders.

Non-coding RNA molecules have emerged as key components of epigenetic regulator networks, with potent capabilities of modulating gene expression in almost every biological and pathogenic process [25–27]. In recent years, roles of long non-coding RNAs have received enormous attention by the research community due to their widespread involvement in multiple functions and mechanisms in changing the target gene expression [16,28]. Of significance is the recent discovery of the distinct roles of lncRNAs in aging in multiple organs and cell types [29–31]. For instance, lncRNA profiles were recently shown to be significantly altered in the substantia nigra in older rat brains, which downregulated the expression of functional protein-encoding genes [32]. By contrast, lncRNA expression alterations have also been linked with the pathogenesis of various human diseases of the vascular system [16]. However, the roles of lncRNAs in directly mediating the aging of vascular tissues remain largely unexplored. The characterization of differentially expressed lncRNAs in the aortas of older rats in this study and a few recent reports addressing lncRNAs in aging vascular cells [18,19,21] indicated the substantial implications of lncRNAs-induced epigenetic regulations in driving vascular aging, which deserve further investigations.

The main biological and pathogenic roles of lncRNAs are mediated by their modulation of functional gene expression through various molecular strategies at the transcriptional and post-transcriptional stages [15]. For a preliminary presentation of the functions of lncRNAs in aging blood vessels, we identified differentially expressed mRNAs in older rat’ thoracic aortas in comparison with young rats, which revealed great changes in the gene expression profiles that were associated with vascular aging. Our functional annotation showed significant enrichments of these differentially expressed genes in multiple biological processes and signaling pathways. For instance, ECM, which is composed of various proteins, such as collagens, fibronectins, polysaccharides, proteoglycans, and glycoproteins, has a large impact on angiogenesis through matrix remodeling and interaction with cell surface receptors [33,34]. In this study, we also found that differentially expressed mRNAs in the aortas were significantly enriched in the ECM–receptor interaction pathway, highlighting the significance of lncRNAs-regulated ECM alterations underlying the vascular aging processes. Furthermore, the phosphorylation and activation of AMPK proteins were previously reported to regulate angiogenesis under hypoxic stress through the modulation of the migration and differentiation of endothelial cells [35]. Here, we also showed significant enrichment of differentially expressed mRNAs in the AMPK signaling pathway, suggesting the great alteration of molecular events accompanying the progression of vascular aging.

MicroRNAs are a large group of non-coding RNAs with a common size of 22 bp, which are known to post-transcriptionally repress gene expression and mediate vascular aging and several vascular pathologies [36–38]. By contrast, the gene expression-regulating functions of microRNAs could be repressed by specific lncRNAs, which are termed as microRNA sponges [23]. Through bioinformatic analysis, we showed that these differentially expressed lncRNAs and mRNAs in the aortas formed a complex interaction network with many microRNA molecules. Among them, AC111292.1 was predicted to sponge mo-miR-29b-3p, which further targets the expression of Col5a2, Col5a1, Col4a1, Col1a1, Col3a1, and Col12a1. Of note, these target genes all encode collagen proteins that serve as main components of the ECM complex. Previous reports have established that collagen synthesis and deposition could critically regulate vessel formation by affecting the survival, proliferation, adhesion, and migration of vascular endothelial cells [34]. These findings indicate that epigenetic regulation of collagen synthesis and ECM remodeling exerts essential impacts on vascular aging and pathogenesis.

Next-generation RNA sequencing and bioinformatics analysis provide comprehensive information of vascular aging from a new perspective and direction. While the method of next-generation RNA sequencing has certain error rate inevitably as all sequencing method have, which could be avoided by further validation. To test the reliability of our RNA sequencing results, we analyzed the expression of six lncRNAs by qRT-PCR method. However, the present study still has several limitations. For example, the expression changes and functions of several lncRNAs and mRNAs need to be further verified in the rats and cell lines. In addition, the prognostic analysis of the differential lncRNAs and mRNAs in old rats need to be further explored. In future studies, to further apply differential lncRNAs and mRNAs for improving vascular aging, detailed animal experiments need to be further performed. According to studies on the effects of exogenous RNA on tissues *in vivo*, we can adopt adeno-associated viruses for gene interference in specific tissues or import exogenous RNA through methods, such as exosome encapsulation. At the same time, we are focusing on whether different foods and drugs have stimulating or inhibiting effects on the corresponding lncRNAs and mRNAs.

## Conclusion

We characterized a large number of lncRNAs and mRNAs that were differentially expressed in thoracic aorta of young and older rats that covered multiple biological processes and signaling pathways, such as ECM–receptor interactions. The differential expression of several collagen genes in rat aorta may be attributed to the sponging of microRNAs by lncRNA AC111292.1. According to these results, we can further explore the treatment of diseases and the delay of aging from a new perspective and direction. In addition, verification of differential lncRNAs in vascular aging can lay a foundation for future research on mechanism in vascular aging.

## Supplementary Material

Supplemental Material

## Highlights

The expression of 36 lncRNAs were significantly altered in old rat’ thoracic aortas.

The expression of 922 mRNAs were significantly altered in old rat’ thoracic aortas.

Significantly alteration of lncRNAs and mRNAs were involved invascular aging.
